# Assessing cytotoxic and antibacterial effects of high dispersion-stable sub-5 nm silver particles fabricated by ionic liquid-mediated electrochemical synthesis

**DOI:** 10.3389/fbioe.2026.1796805

**Published:** 2026-04-21

**Authors:** Fen Zhang, Lihao Ou, Jining Shao, Meng Gu, Haiyang Jia

**Affiliations:** 1 School of Food and Bioengineering, Xuzhou University of Technology, Xuzhou, China; 2 School of Chemistry and Chemical Engineering, Southeast University, Nanjing, China; 3 School of Physics and New Energy, Xuzhou University of Technology, Xuzhou, China

**Keywords:** antibacterial activity, cytotoxicity, electrochemical preparation, ionic liquid, silver nanoparticles

## Abstract

Exploring nanoscale metal particles with high colloidal stability and antibacterial activity is critical for advancing biomedicine in the context of antibiotic resistance. In this study, ultra-small silver nanoparticles (Ag-NPs) with sub-5 nm were synthesized in a one-step electrochemical route in an ionic-liquid-containing electrolyte. The resulting colloid of Ag-NPs remained for more than 400 days without precipitation and ultraviolet-visible spectral shift. Dynamic light scattering gives an average hydrated particle diameter of 2.8 nm and a Zeta potential of −0.6 mV. Cell viability and cytotoxicity results showed high cell compatibility of Ag-NPs at ≤ 4 μg/mL and obvious cell death at 16 μg/mL. The antibacterial potency of Ag-NPs was evaluated using the Oxford cup method and the broth dilution test. The minimum inhibitory concentration of Ag-NPs against the Gram-positive bacteria *Bacillus subtilis* and *Staphylococcus aureus* was 4 μg/mL, whereas that for the Gram-negative species *Escherichia coli* was as low as 2 μg/mL. The minimum bactericidal concentration of Ag-NPs against three types of bacteria was 8 μg/mL. The long-term colloidal stability, acceptable cytotoxicity, and broad-spectrum bactericidal activity of Ag-NPs were demonstrated. These ultra-small nanoscale metal particles hold great potential for antibacterial applications like wound care and antibacterial coating.

## 1 Introduction

Nanoscale metal-based bacteriostatic agents were regarded as those that could bypass or mitigate the resistance mechanisms evolved against conventional antibiotics (Xu et al., 2023; Miller et al., 2013; AlQurashi et al., 2025; Altun et al., 2021). Among metallic candidates, silver has remained a benchmark for broad-spectrum biocidal activity since antiquity (Gołuński et al., 2024). Silver nanoparticles (Ag-NPs) have garnered sustained attention due to their broad-spectrum bactericidal activity, low propensity for inducing resistance, and versatility in surface functionalization (Miller et al., 2013; Zhao et al., 2022; Jia et al., 2024; González et al., 2023; Nie et al., 2023; Ali et al., 2025). However, the practical translation of Ag-NPs into disinfectants, coatings, or wound-care formulations is still hampered by two persistent challenges: (i) uncontrolled particle growth during synthesis that often yields polydisperse species with diminished bioactivity; (ii) poor colloidal stability in physiological media, which triggers aggregation, sedimentation, and premature loss of antibacterial efficacy (Ali et al., 2025; Zulfiqar et al., 2024; Magdy et al., 2024; Vadakkan et al., 2024). Contemporary deployment of Ag in nanoparticulate form still faces critical bottlenecks that originate from the synthesis step itself. Traditional chemical reduction routes, whether biological extract-, borohydride-, citrate-, or polyol-mediated, afford rapid nucleation but offer limited kinetic control over the growth phase, resulting in Ag nanoparticles predominantly larger than 20 nm (Deshpande et al., 2021; Khatoon et al., 2023; Ahmad et al., 2024; Jacob et al., 2024; Sganzerla et al., 2023). Consequently, the development of reproducible and scalable protocols that can stably confine ultra-small silver particles in the sub-10 nm or even sub-5 nm regime while simultaneously imparting long-term colloidal stability is regarded as a critical step toward next-generation nanosized biological reagents (Bélteky et al., 2021; Zhang et al., 2022).

Electrolytic synthesis offered an attractive alternative, such as water-soluble sub-10 nm iron oxide and sub-10 nm silver colloids (Jia et al., 2024; Jia et al., 2021a; Jia et al., 2023a; Jia et al., 2023b). By separating silver ion generation (anodic dissolution) from the subsequent reduction (cathodic surface or bulk reaction), the process allowed the solvated Ag^+^ to nucleate continuously under mild electrochemical conditions. Theoretically, by fine-tuning current density and electrolyte composition, and with the assistance of steric or electrostatic stabilizers, the growth stage could be quenched at a specific scale (Jia et al., 2024). Realistically, due to the parasitic oxygen evolution derived from water decomposition and their participation in the reaction, classical aqueous electrochemical systems for preparing ultra-small Ag-NPs suffer from progressive agglomeration and impurities. These unfavorable factors, combined with the regulation of pH levels, broaden the size distribution and shorten the shelf life. In severe cases, once the current is interrupted, the sol often evolves into a polydisperse mixture within hours. The preparation of Ag-NPs colloid by electrolysis in an organic electrolyte has been proven to be a feasible approach. The design of the electrolyte is a crucial aspect of this process. In the previous report, sub-10 nm silver colloids were prepared by electrolysis in an organic electrolyte consisting of a charge carrier (silver nitrate), a stabilizer (polyvinylpyrrolidone), and an organic solvent (mixture of protonic ethanol and non-protonic dimethylformamide). The prepared colloid was well-dispersed and stable (21 days) (Jia et al., 2024).

Generally, for nanoscale silver dispersions, the biological activity is inversely proportional to particle size and directly proportional to dispersion stability (Bélteky et al., 2021; Zhang et al., 2022). Building on the existing protocol, tailoring the electrolyte composition might render the electrochemical synthesis of sub-5 nm silver colloids a viable strategy. Essentially, the charge carrier and stabilizer are necessary, and they should be electrochemically inert and insensitive to free silver ions. Due to negligible vapor pressure, wide electrochemical window, and tunable ion-ion interactions, ionic liquids and their analogs (e.g., deep eutectic solvents) have recently emerged as multifunctional electrolytes or key components for electrochemical synthesis (Jia et al., 2021b; Coskun et al., 2024; Jia et al., 2025; Liu et al., 2023). Given their compatibility with silver ions, the imidazolium-nitrate ionic liquid deserves attention as a substituent (Kowsari and Torabi, 2020; Abe and Kishimura, 2022; Abe et al., 2024). Moreover, the higher solubility of ionic liquids enables them to act as charge carriers with conductivity superior to that of inorganic nitrates in organic solvents. A single-component aprotic polar organic solvent with a low saturated vapor pressure was preferred over a multi-component mixture to ensure chemical inertness and reaction stability during the electrolytic process (Jia et al., 2021b). Classical nonionic N-vinyl amide polymers remain potential stabilizers due to their joint action of steric hindrance, secondary electrostatics, and surface complexation/capping (Yang et al., 2021).

The present investigation was therefore designed to fill this knowledge gap by developing a one-pot, low-energy electrochemical method that continuously produces ultra-small sub-5 nm Ag-NPs in a 1-butyl-3-methylimidazolium nitrate/dimethyl formamide/polyvinylpyrrolidone electrolyte. The size distribution and colloidal stability of Ag-NPs were characterized systematically. The cytotoxicity was assessed qualitatively and quantitatively based on fluorescent and spectrophotometric detection using HepG2 (human hepatocellular carcinoma) and A549 (human non-small cell lung cancer) cell lines. Further, the antibacterial potency of Ag-NPs against both Gram-positive and Gram-negative bacteria was tested by the classic Oxford cup method and the broth dilution assay, whereby the minimum inhibitory concentration (MIC) and minimum bactericidal concentration (MBC) were quantitatively determined.

## 2 Experimental section

### 2.1 Preparation of Ag-NPs

An electrolyte was obtained by dissolving polyvinylpyrrolidone (guaranteed reagent; Sinopharm Chemical Reagent, China; 4 mg/mL) and 1-butyl-3-methylimidazolium nitrate (95%; Macklin Inc., Shanghai, China; 2 mg/mL) in dimethyl formamide (99.5%, analytical reagent; Shanghai Lingfeng Chemical Reagent, China). Two identical silver plates were placed symmetrically in the electrolyte, and the electrolytic cell was maintained at 45 °C by an oil bath. A constant direct-current density of 2.5 mA/cm (relative to the cathode’s submerged area) was supplied by a stabilized power supply (GPS−4030; Good Will Instrument, China). Once the electrochemical preparation was completed, the electrolyte was subjected to high-speed centrifugation at 10,000 rpm for 5 min. Approximately half of the supernatant after centrifugation was then filtered successively through needle-type filters with precision ratings of 0.45 μm and 0.22 μm. The obtained silver colloid was stored in glass bottles in a light-proof environment at room temperature. The quantitative concentration of the colloid was determined by a plasma mass spectrometer (Agilent ICP−MS 7700). It should be noted that the silver nanoparticles in the colloid were standardized to a concentration of 2 mg/mL for subsequent experiments. This adjustment was achieved by diluting the filtered electrolyte with purified water (Wahaha®). Samples were maintained at room temperature under light protection throughout the year.

### 2.2 Characterization

The ultraviolet–visible (UV–Vis) spectrum of the colloid was measured on a Shimazu UV-2600 spectrophotometer. The crystalline state of the nanoparticles in the colloid was identified using high-resolution transmission electron microscopy (HR-TEM, FEI Tecnai G2 F20). The Zeta potential and hydrodynamic diameter (via dynamic light scattering) of the colloid were measured using a Zetasizer Nano ZS90 after dilution with pure water.

### 2.3 Cytotoxicity testing

#### 2.3.1 Materials and reagents

Fetal bovine serum (FBS), Dulbecco’s Modified Eagle Medium (DMEM), fluorescein diacetate (FDA), propidium iodide (PI), dimethyl sulfoxide (DMSO), and 3-(4,5-dimethylthiazol-2-yl)-2,5-diphenyltetrazolium bromide (MTT) were purchased from Jiangsu KeyGEN BioTECH Crop., Ltd. (Nanjing, China). All other chemical reagents, unless specifically stated, were of analytical purity and were purchased from local commercial suppliers. All aqueous solutions were prepared using ultra-purified water supplied by a Milli-Q system (Millipore®).

#### 2.3.2 Cell culture

HepG2 and A549 cells were obtained from the Chinese Academy of Sciences (Shanghai, China). In a humidified atmosphere containing 5% CO_2_, these cells were cultured in DMEM supplemented with 10% FBS, 100 units/mL penicillin, and 100 μg/mL streptomycin at 37 °C. Before use, the cells were first harvested by trypsinization with 0.25% trypsin at 37 °C. The trypsinization was terminated by adding fresh supplemented DMEM. Next, the cell suspension was centrifuged at 1,000 rpm for 3 min, followed by a supernatant removal, the cells were finally resuspended in fresh supplemented DMEM for use.

#### 2.3.3 Cell viability evaluation by FDA/PI double-staining

The viability of cells was qualitatively assessed using the FDA/PI double-staining (Zhang et al., 2018; Zhang et al., 2025; Zhang et al., 2020). The effect of the nanoparticle dose on the viability was investigated as follows. HepG2 and A549 cells, at a density of 5 × 10^4^ cells/mL, were seeded in 12-well plates and cultured for 24 h. The culture medium was discarded and the cells were washed three times with phosphate-buffered saline (PBS, pH 7.4). Next, Ag-NPs were added at various concentrations (0.5, 1, 2, 4, 8, and 16 μg/mL in the medium). After a 24 h incubation, the culture medium containing the Ag-NPs was discarded, and the cells were washed three times with PBS. The cells were then incubated with an FDA/PI staining solution (10 μg/mL) at 37 °C for 5 min, followed by rinsing with PBS. A fluorescence microscope (FV3000, Olympus, Japan) was used for imaging. During the experiment, a blank control group was included.

The effect of exposure time on cell viability was investigated too. HepG2 and A549 cells were seeded and cultured as above. The culture medium was discarded and the cells were washed thrice with PBS. Next, Ag-NPs at 4 μg/mL were added for the treatment. After incubating for 2, 12, 24, 36, and 48 h, the culture medium containing Ag-NPs was discarded. The samples were then washed three times with PBS, and subsequently incubated with FDA/PI staining solution (10 μg/mL) at 37 °C for 5 min, followed by washing with PBS. A blank control group was included in the experiment.

#### 2.3.4 Cytotoxicity assessment by MTT assay

The cytotoxicity of the Ag-NPs was quantitatively assessed using the standard MTT method (Wang et al., 2011). In the investigation of dose impact on cytotoxicity, HepG2 and A549 cells were seeded in 96-well plates at a cell density of 5 × 10^4^ cells/mL (200 μL/well), respectively. After a 24 h cultivation, the cells were incubated in fresh culture media containing Ag-NPs at different doses (0.5, 1, 2, 4, and 6 μg/mL) for 24 h. The culture medium was replaced by MTT solution (5 mg/mL, 20 μL/well) and fresh culture medium (150 μL/well), followed by further culture for 4 h. The supernatant was discarded, and 200 μL of DMSO was added to the wells. The plates were then placed in a shaking incubator (SKY-100 B, Sukun Industrial, Shanghai, China) for 10 min.

To investigate the impact of nanoparticle exposure time on cytotoxicity, HepG2 and A549 cells at a density of 5 × 10^4^ cells/mL were seeded in the respective 96-well plates. After a 24 h cultivation, the cells were incubated with Ag-NPs (4 μg/mL in fresh culture medium) for various times (2, 12, 24, 36, and 48 h). Next, the culture medium was replaced by MTT solution (5 mg/mL, 20 μL/well) and fresh culture medium (150 μL/well), followed by further culture for 4 h. The supernatant was discarded, and 200 μL of DMSO was added to the wells. The plates were then placed in a shaking incubator (SKY-100 B, Sukun Industrial, Shanghai, China) for 10 min.

The absorbance (A) of each well was measured at 490 nm using a microplate reader (model 680, BIO-RAD). Cell viability was then calculated as follows (Equation 1):

$$\text{Cell viability }\left(\%\right)=\frac{A_{s}}{A_{c}}\times 100$$ (1)

where *A*s and *A*c are the absorbance values of the sample (Ag-NPs) group and control group, respectively. Each experiment was repeated at least three times. The background absorbance control was set too.

### 2.4 Antibacterial test

#### 2.4.1 Preparation of bacterial suspension

Stock cultures of *E. coli* (*Escherichia coli*), *B. subtilis* (*Bacillus subtilis*), and *S. aureus* (*Staphylococcus aureus*) were inoculated separately into Luria-Bertani (LB) liquid medium (Sigma-Aldrich, United States of America) and cultured at 37 °C for 24 h with a shaking speed of 120 rpm. Then, the bacterial suspension was diluted with LB medium to a concentration of 1 × 10^8^ CFU/mL (colony-forming units per mL) for further use.

#### 2.4.2 Bacteriostatic zone assay

The antibacterial activity of Ag-NPs against *E. coli*, *S. aureus*, and *B. subtilis* was evaluated using the Oxford cup method (Mohammed et al., 2023; Zhang et al., 2024). Different bacterial solutions mentioned above were applied onto culture dishes containing solid culture medium. Four Oxford cups (outer diameter: 8 mm) were evenly positioned on each culture medium. Each cup received 100 μL of the medicinal liquid. The experimental setup included an antibiotic mixture (penicillin: 100 units/mL; streptomycin: 100 μg/mL) as the positive control (labeled I), sterile normal saline as the negative control (labeled II), and Ag-NPs colloids at concentrations of 10 and 20 μg/mL as the experimental groups (labeled III and IV, respectively). The culture dishes were incubated at 37 °C for 20 h. After incubation, the diameters of the inhibition zones were measured using the cross method. Each experiment was performed at least three times.

#### 2.4.3 Determination of antibacterial ability

The antibacterial efficacy of Ag-NPs was assessed using the standard broth dilution method according to the CLSI guidelines (Behravan et al., 2019; Li et al., 2023). Firstly, multiple autoclaved test tubes were prepared in a sterile environment. To each tube, 5 mL of nutrient broth and 10 μL of bacterial suspension at 2 × 10^8^ CFU/mL were added. Then, the Ag-NPs colloids with different concentrations were added, respectively. The positive control consisted of nutrient broth inoculated with bacteria, and the negative control contained nutrient broth without bacteria. After shaking at 37 °C for 24 h, the experimental results were observed. If turbidity appears, record the result as positive (+). If the test tube remains clear, record it as negative (−). The lowest concentration of Ag-NPs in the negative test tube is the MIC. 100 μL of solution was taken from each negative test tube and spread onto different types of solid culture media plates. The plates were inverted and incubated at 37 °C for 24 h. The growth of the colonies was visually inspected. Plates with colony counts of equal or greater than 5 were designated as positive (+), while those with fewer than 5 colonies were designated as negative (−). The lowest concentration of Ag-NPs corresponding to the negative plates was defined as the MBC. Each experiment was performed at least three times.

### 2.5 Statistical analysis

Software Microsoft Excel (Microsoft) was used to conduct data statistical analysis. All quantitative data were shown as mean ± standard deviation. Statistical comparisons of means were conducted using one-way analysis of variance. *P < 0.05 was used as the criterion for statistical significance.

## 3 Results and discussion

### 3.1 Phase, size, and dispersion analysis

**FIGURE 1:**
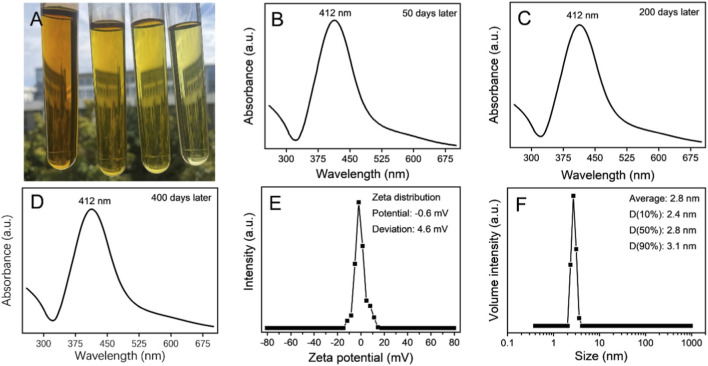
Characterization of Ag-NPs in the colloid. **(A)** Actual pictures of the silver colloids stored for 400 days at various concentrations (left to right: 200, 100, 50, and 25 μg/mL). **(B–D)** UV-visible spectra of the colloid after storage for 50 days **(B)**, 200 days **(C)**, and 400 days **(D)**. Zeta potential data **(E)** and hydrated particle size distribution **(F)** of the colloid at the 400th day.

The prepared silver colloids are shown in Figure 1A. The solution-like appearance indicates nanoscale particle size distribution and excellent dispersibility. As the concentration increases, the displayed color transitions from yellow to black. The UV−Vis spectra of the colloids are shown in Figures 1B−D. The absorption peak centered at 412 nm is a typical characteristic of Ag-NPs (Hamid et al., 2023; Wang and Wei, 2022; Nassar et al., 2024). During 400 days of storage, the spectral absorption peaks of the colloid remain highly similar, demonstrating the high stability of the colloid dispersion. The stable dispersion of the colloid reflects the ultra-small size of the nanoparticles. The zeta-potential distribution of the colloid at the 400th day was centered at −0.6 mV with a standard deviation of 4.6 mV (Figure 1E). The prepared colloid contains nitrate anions and their corresponding cations, and the synthesized silver nanoparticles can maintain dispersion stability in the medium solutions. The absolute value of the Zeta potential of the colloid is no more than 5 mV, indicating that the dispersion of nano-silver in the colloid is based on the steric hindrance stabilization rather than the electrostatic effect. This steric hindrance is likely caused by the long molecular chains of PVP, suggesting that PVP at least functions as a stabilizer. Fundamentally, PVP is widely used in the preparation of silver nanoparticles and their colloids. (Jia et al., 2024; Nie et al., 2023; Magdy et al., 2024; Vadakkan et al., 2024). In addition, the results of the periodical detections (Figure 1B−F; Supplementary Figure S1) verified the stability of the colloid over 400 days.

**FIGURE 2:**
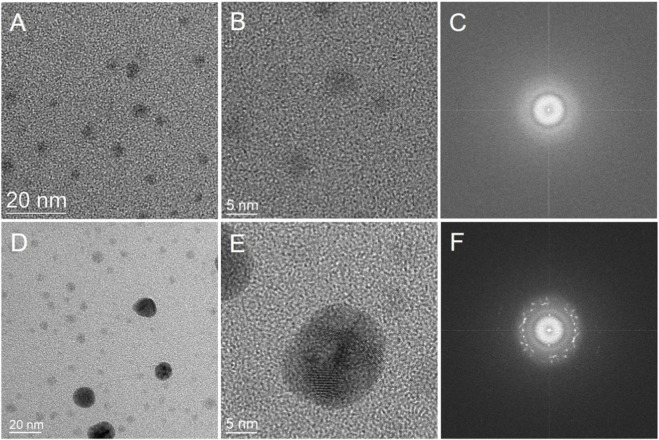
TEM images **(A,D)**, HR−TEM images **(B,E)**, and Fast Fourier Transform images **(C,F)** of the Ag-NPs in the colloid.

Dynamic light scattering analysis revealed a statistically average particle size of 2.8 nm and a 90% particle size (D90) of 3.1 nm (Figure 1F) of the colloid at the 400th day. These distributions were visually confirmed by TEM observations (Figure 2A). The Ag-NPs were so small that it was difficult for them to form the distinct crystal structure, exhibiting amorphous properties (Shao et al., 2024; Tong et al., 2024). This was reflected in the absence of lattice fringes in the HR-TEM record (Figure 2B) and diffraction spots in the corresponding Fast Fourier Transform pattern (Figure 2C). Some slightly larger particles, ranging in size from 10 to 15 nm, were observed in TEM observations (Figure 2D). These larger nanoparticles exhibit lattice fringes in the HR−TEM recording (Figure 2E) and diffraction spots in the corresponding Fast Fourier Transform image (Figure 2F), indicating the crystalline structure (Jia et al., 2024; Martínez-Higuera et al., 2021; Sankareswari et al., 2022). Totally, the ultra-small particle size distribution and stable dispersion state of these Ag-NPs form the basis for achieving the expected biological effects.

### 3.2 Cytotoxicity evaluation

#### 3.2.1 Fluorescence characterization of cell viability

**FIGURE 3:**
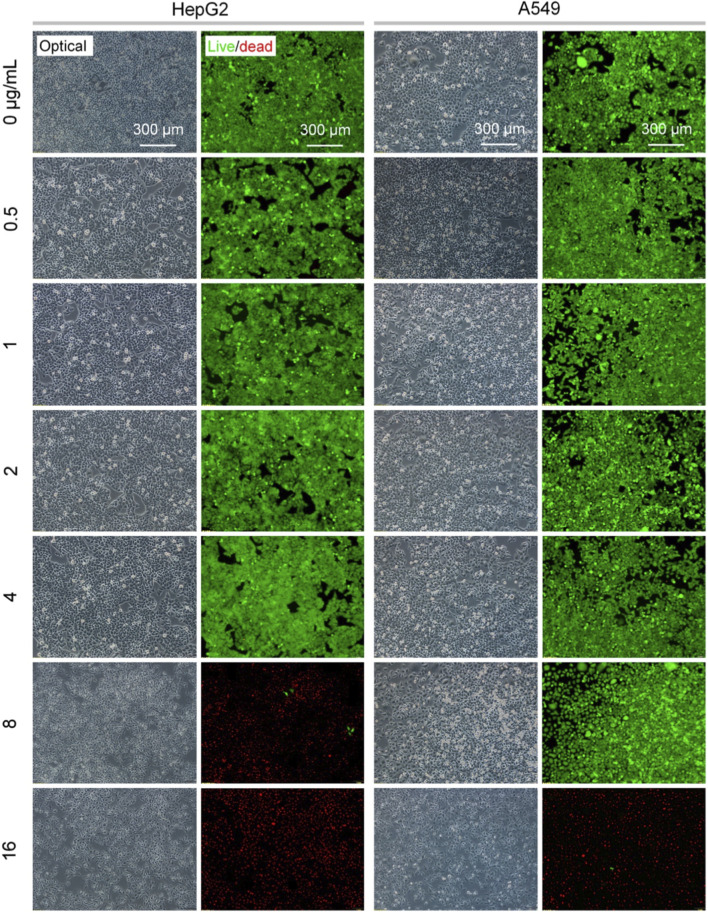
Viability of HepG2 and A549 cells after treatment with different concentrations of Ag-NPs (0–16 μg/mL) for 24 h. Live/dead (green/red) cells were visualized by FDA/PI staining.

**FIGURE 4:**
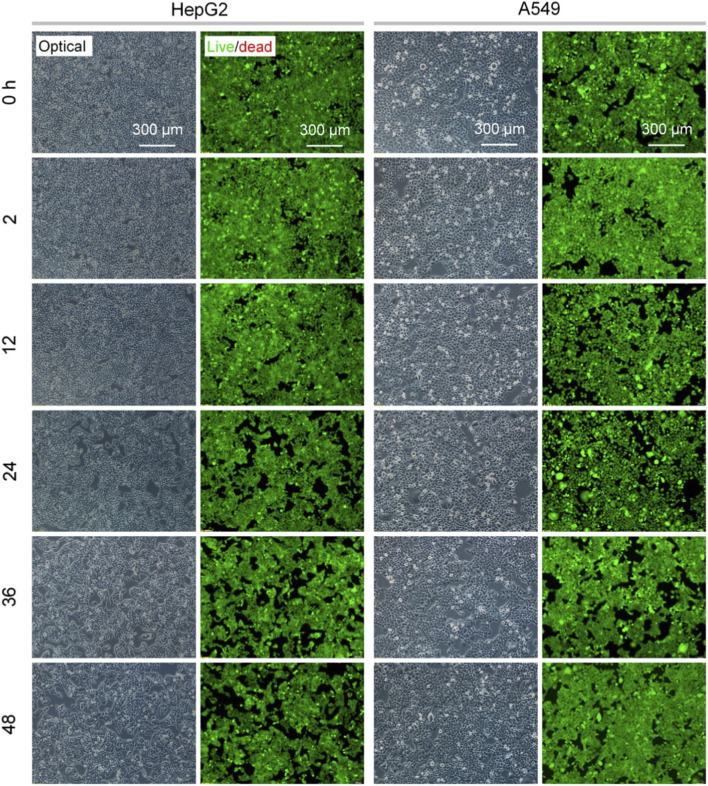
Viability of HepG2 and A549 cells treated with 4 μg/mL of Ag-NPs for various treating times (0–48 h).

To assess the cell compatibility and cytotoxicity of the fabricated Ag-NPs, cell viability was measured using the FDA/PI double fluorescence staining. HepG2 and A549 cells being commonly used for assessing nanotoxicity were selected here (Akter et al., 2018; Singh and Ramarao, 2012). The results (Figures 3, 4; Supplementary Figures S2, S3) showed that the viability was negatively correlated with the concentration of nanoparticle treatment. This resultant tendency was similar to the previous reports involving Ag-NPs (Sankareswari et al., 2022; Gliga et al., 2014). Almost no dead cells (red fluorescence dots) were observed in HepG2 and A549 cultures after the 24 h treatment at 0–4 μg/mL (Figure 3; Supplementary Figure S2). A significant number of HepG2 cells died when the treating dose increased up to 8 μg/mL, while A549 cells remained highly viable. When exposed to a 16 μg/mL of Ag-NPs, all cells exhibited a significant apoptotic response within 24 h (Figure 3). Furthermore, a long-term (48 h) stimulation of Ag-NPs at 4 μg/mL did not cause a significant increase in cell death of HepG2 and A549 cultures (Figure 4; Supplementary Figure S3). These results implied that the Ag-NPs at the dose of 4 μg/mL were highly compatible with cells and presented the cytotoxic effect for both cell lines when the dose reached 16 μg/mL.

#### 3.2.2 Cytotoxicity quantification

**FIGURE 5:**
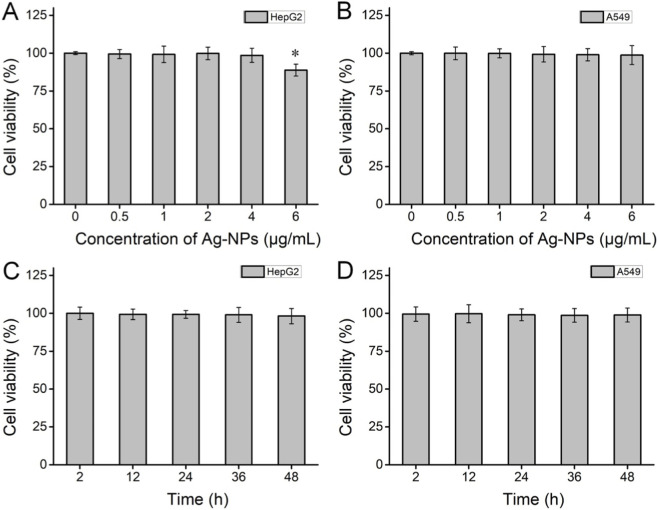
Quantitative cytotoxicity using the MTT assay. **(A,B)** Viability of the HepG2 **(A)** and A549 **(B)** under a concentration gradient (o to 6 μg/mL). **(C,D)** Viability of the HepG2 **(C)** and A549 **(D)** under a time gradient with the treatment of 4 μg/mL Ag-NPs. Error bar: ±SD (n = 5) *P < 0.05.

Next, the cytotoxic effect of Ag-NPs on HepG2 and A549 cells was quantitatively assessed using the standard MTT method. The results (Figures 5A,B) showed that the two types of cells exhibited adequate tolerance to the synthesized Ag-NPs (0.5–6 μg/mL). The cell survival rates totally remained over 98.5% after the 24-h treatment of Ag-NPs at different doses except 6 μg/mL for HepG2 cells (88.7%). These quantitative achievements were consistent the aforementioned outcomes (Figure 3). After treated with the 4 μg/mL Ag-NPs for different incubation time (2, 12, 24, 36, and 48 h), we noticed that the viability of both HepG2 and A549 cells was at least 98.2% (Figure 5C), suggesting high cell compatibility (Figures 5C,D).

The assessments by the FDA/PI double staining and MTT assay showed differences in the Ag-NPs-induced responses of HepG2 and A549 cell lines. These two types of cells originate from cancerous tissues of the liver and the lung, respectively, which might be one of the key factors contributing to the observed difference in cytotoxicity (Akter et al., 2018; Gliga et al., 2014; Singh and Ramarao, 2012). Importantly, the results above verified that the fabricated sub-5 nm Ag-NPs with high colloidal stability were fairly compatible with mammalian cells when the dose was not exceeding 4 μg/mL, as well as were significantly cytotoxic at the dose of over 16 μg/mL. This implies the possible antibacterial applications of low-cytotoxic Ag-NPs at the specific dose in biomedicine.

### 3.3 Antibacterial activity

#### 3.3.1 Bacteriostatic zone analysis

**FIGURE 6:**
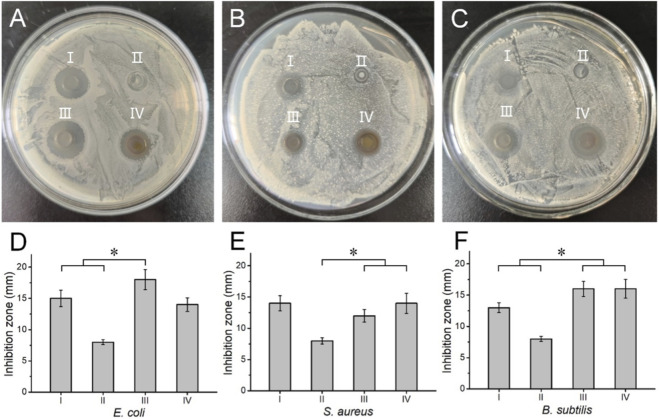
Antibacterial testing using the Oxford cup method. **(A–C)** Photographs for Ag-NPs against *Escherichia coli* **(A)**, *Staphylococcus aureus* **(B)**, and *Bacillus subtilis* **(C)**. **(D–E)** Inhibition zone analysis of *Escherichia coli* **(D)**, *Staphylococcus aureus* **(E)**, and *Bacillus subtilis* **(F)**. Group I used a mixture of penicillin and streptomycin as the positive control, and Group II used normal saline as the negative control. Groups III and IV were treated with silver colloids at the concentration of 10 and 20 μg/mL, respectively, as the experimental groups. Error bar: ±SD (n = 3) *P < 0.05.

The antimicrobial efficacy of Ag-NPs against various microorganisms supports their effective use as an antibacterial agent. The Oxford cup method was employed to evaluate the antibacterial activity of the Ag-NPs against the Gram-positive bacteria *S. aureus* and *B. subtilis*, as well as the Gram-negative bacterium *E. coli*. For comparison, normal saline was used as a negative control. A mixture of penicillin and streptomycin antibiotics was used as a positive control. The inhibition zones caused by Ag-NPs against *E. coli* (Figure 6A), *S. aureus* (Figure 6B), and *B. subtilis* (Figure 6C) were clearly observed, along with the corresponding measured sizes (diameter) of the inhibition zones plotted in Figures 6D–F. This demonstrates that Ag-NPs have an antibacterial feature. Relative to both controls, Ag-NPs can significantly enhance the antibacterial efficacy against *E. coli* (Figure 6A) and *B. subtilis* (Figure 6C). At a concentration of 10 μg/mL, Ag-NPs produced the largest inhibition zones against *E. coli* (18 mm) and *B. subtilis* (16 mm) (Figures 6D,F), being obviously larger than the positive control. Comparatively, the antibacterial property of Ag-NPs against *S. aureus* was similar to that of the latter (penicillin and streptomycin antibiotics) (Figures 6B,E). These results indicated that the antibacterial ability of the Ag-NPs against Gram-negative bacteria was higher than that against Gram-positive bacteria. This difference might stem from the thicker peptidoglycan-rich cell wall of Gram-positive bacteria, which hinders the penetration of Ag-NPs and thus alters their antibacterial mechanism (Pazos-Ortiz et al., 2017).

We demonstrated that the antibacterial activity of the Ag-NPs against the three bacteria was comparable to or even exceeded that of antibiotics (positive control), indicating the effectiveness of Ag-NPs and their ability to achieve efficient and broad-spectrum bactericidal effects against both Gram-negative and Gram-positive bacteria. Our antibacterial results of sub-5 nm Ag-NPs were consistent with the previous outcomes of the larger Ag-NPs against *E. coli*, *S. aureus,* and *B. subtilis* (Guzman et al., 2012; Alsamhary, 2020). The mechanism of antibacterial activity of Ag-NPs can be described as follows: Ag-NPs release silver ions, which disrupt the cell wall and cytoplasm. In addition, they induce the production of reactive oxygen species, thereby inhibiting respiratory enzymes and halting the synthesis of adenosine triphosphate (Mohammed et al., 2023).

#### 3.3.2 Antibacterial ability analysis

**FIGURE 7:**
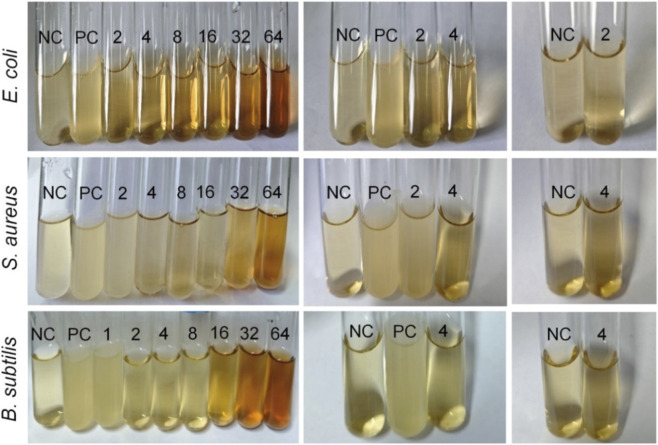
MIC analysis. Representative images showing bacterial growth inhibition in *Escherichia coli*, *Staphylococcus aureus*, and *Bacillus subtilis* at different Ag-NP concentrations (2–64 μg/mL). NC: Negative control; PC: Positive control.

The standard broth dilution assay was further employed to determine the MIC and MBC of Ag-NPs against the standard strains of *E. coli*, *S. aureus*, and *B. subtilis*. The MIC assay results are shown in Figure 7. Generally, the higher the transparency of the culture medium in the glass test tube, the fewer the number of surviving bacteria, indicating that the antibacterial ability of the Ag-NPs at the tested dose is more prominent or the antibacterial effect is positive. The collected MIC and MBC values are summarized in Supplementary Tables S1, S2, respectively. For Ag-NPs against *E. coli*, the MIC was determined to be 2 μg/mL, while the MIC for *S. aureus* and *B. subtilis* were 4 μg/mL. It implied that *E. coli* was more susceptible to Ag-NPs than Gram-positive bacteria, which aligned with the antibacterial test results obtained using the Oxford Cup diffusion method.

**FIGURE 8:**
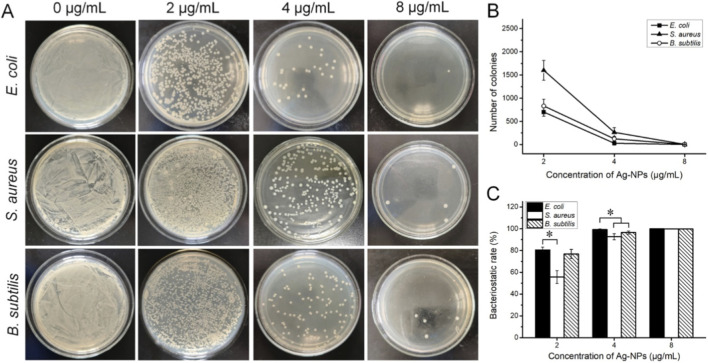
MBC analysis. **(A)** Representative colony plates of *Escherichia coli*, *Staphylococcus aureus*, and *Bacillus subtilis* treated with Ag-NPs at 2, 4, and 8 μg/mL. **(B)** Quantified colony counts of three bacterial strains. **(C)** Bacterial inhibition rates for tested strains. Error bar: ±SD (n = 5) *P < 0.05.

As shown in Figure 8A, the number of colonies declined significantly when the Ag-NPs concentration increased from 2 to 8 μg/mL. This indicated a progressive improvement in antibacterial efficacy. The lowest concentration of Ag-NPs causing almost no bacteria survival (the number of colonies did not exceed 5, Figure 8B) was 8 μg/mL, thus determining as the MBC value for all three bacteria. Further, we found that the bactericidal effect of low-dose Ag-NPs (e.g., 4 μg/mL) against *E. coli* is stronger than that against *S. aureus* and *B. subtilis* (Figure 8C). The antibacterial activity of Ag-NPs depends on the composition and thickness of the bacterial cell wall. Gram-positive bacteria are less sensitive to Ag-NPs, mainly because their cell walls are thicker and contain higher amounts of peptidoglycan (Pazos-Ortiz et al., 2017). The thick cell wall of Gram-positive bacteria, along with the negatively charged peptidoglycan, can trap silver ions within the cell wall. In addition, Gram-negative bacteria possess lipopolysaccharides in their outer membrane and the negative charge of these lipopolysaccharides enhances the attachment of Ag-NPs and renders the bacteria more susceptible to the antimicrobial agent (Dakal et al., 2016; Abbaszadegan et al., 2015).

## 4 Conclusion

In summary, we fabricated ultra-small sub-5 nm Ag-NPs using a facile electrochemical synthesis method in an ionic liquid-based organic electrolyte. The resulting silver colloid remained highly stable for over 400 days with an average particle size of 2.8 nm and near-neutral Zeta potential. Ag-NPs presented high cell compatibility at ≤ 4 μg/mL and considerable cytotoxicity at 16 μg/mL for HepG2 and A549 cells. The broad-spectrum bactericidal activity of Ag-NPs was validated with the MIC of 2 μg/mL for *E. coli* and 4 μg/mL for *S. aureus* and *B. subtilis*, as well as the MBC of 8 μg/mL for all tested strains. These sub-5 nm Ag-NPs offer a promising balance of stability, biocompatibility, and antimicrobial efficacy. We envision that this ultra-small nanomaterial has considerable potential for biomedical and antibacterial applications, such as environmental disinfection and external packaging protection.

## Data Availability

The original contributions presented in the study are included in the article/Supplementary Material, further inquiries can be directed to the corresponding authors.
