# Supplementary material for: Assessing cytotoxic and antibacterial effects of high dispersion-stable sub-5 nm silver particles fabricated by ionic liquid-mediated electrochemical synthesis
Source: Front Bioeng Biotechnol. 2026 Apr 21;14:1796805. doi: 10.3389/fbioe.2026.1796805 (PMC13139332; doi:10.3389/fbioe.2026.1796805)
Supplement: Supplementary file 1 [file DataSheet1.pdf]

Supporting information

Assessing cytotoxic and antibacterial effects of high dispersion-stable sub-5 nm silver particles fabricated by ionic liquid-mediated electrochemical synthesis

Fen Zhang[1,2,]*, Lihao Ou[1], Jining Shao[1], Meng Gu[1], Haiyang Jia[3,]*

[1] *School of Food and Bioengineering, Xuzhou University of Technology, Xuzhou 221018, China*

[2] *School of Chemistry and Chemical Engineering, Southeast University, Nanjing 211189, China*

[3] *School of Physics and New Energy, Xuzhou University of Technology, Xuzhou 221018, China*

*Corresponding authors and E-mail addresses: zhangfen@xzit.edu.cn (F. Zhang), H. Jia (jsz@xzit.edu.cn)

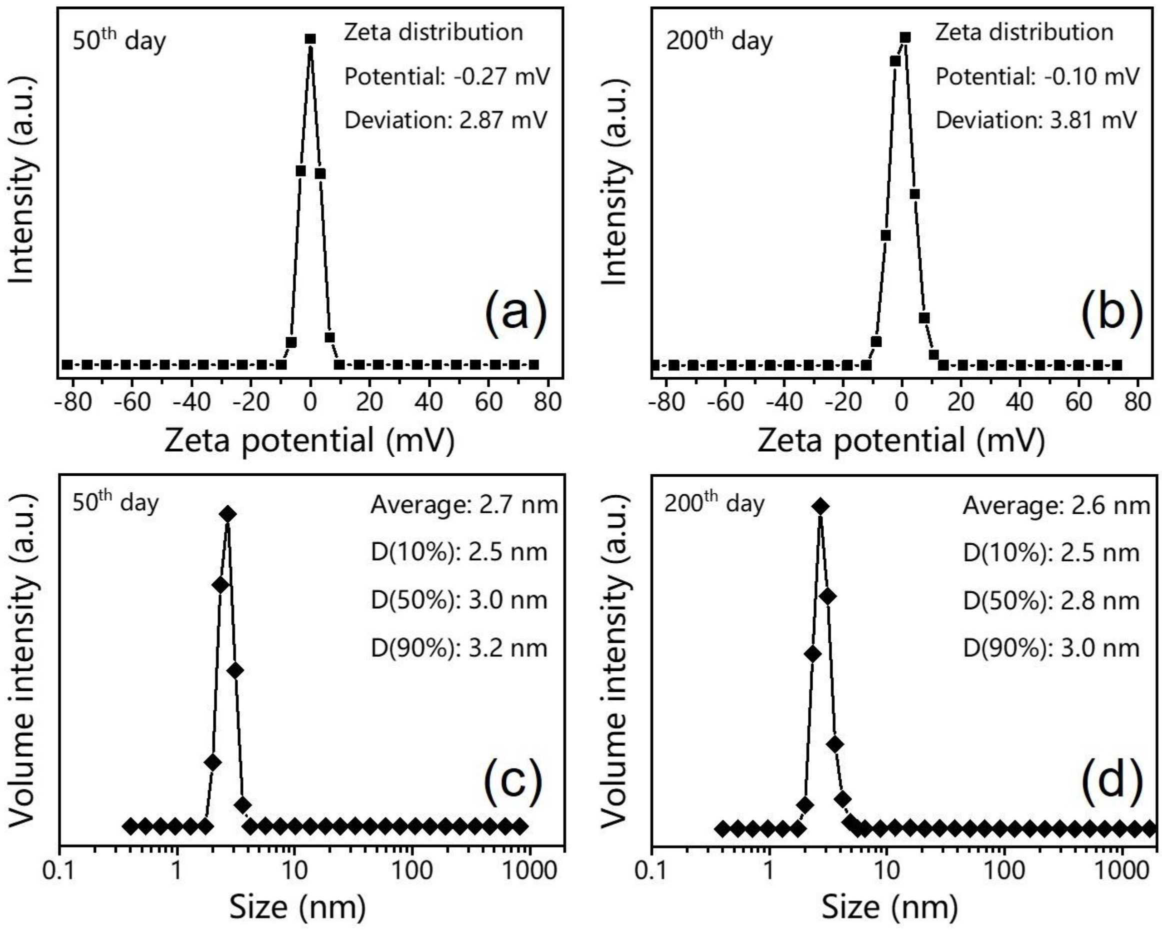

Figure S1. Zeta potential data (a, b) and hydrated particle size distribution (c, d) of the colloid after holding for 50 and 200 days, respectively.

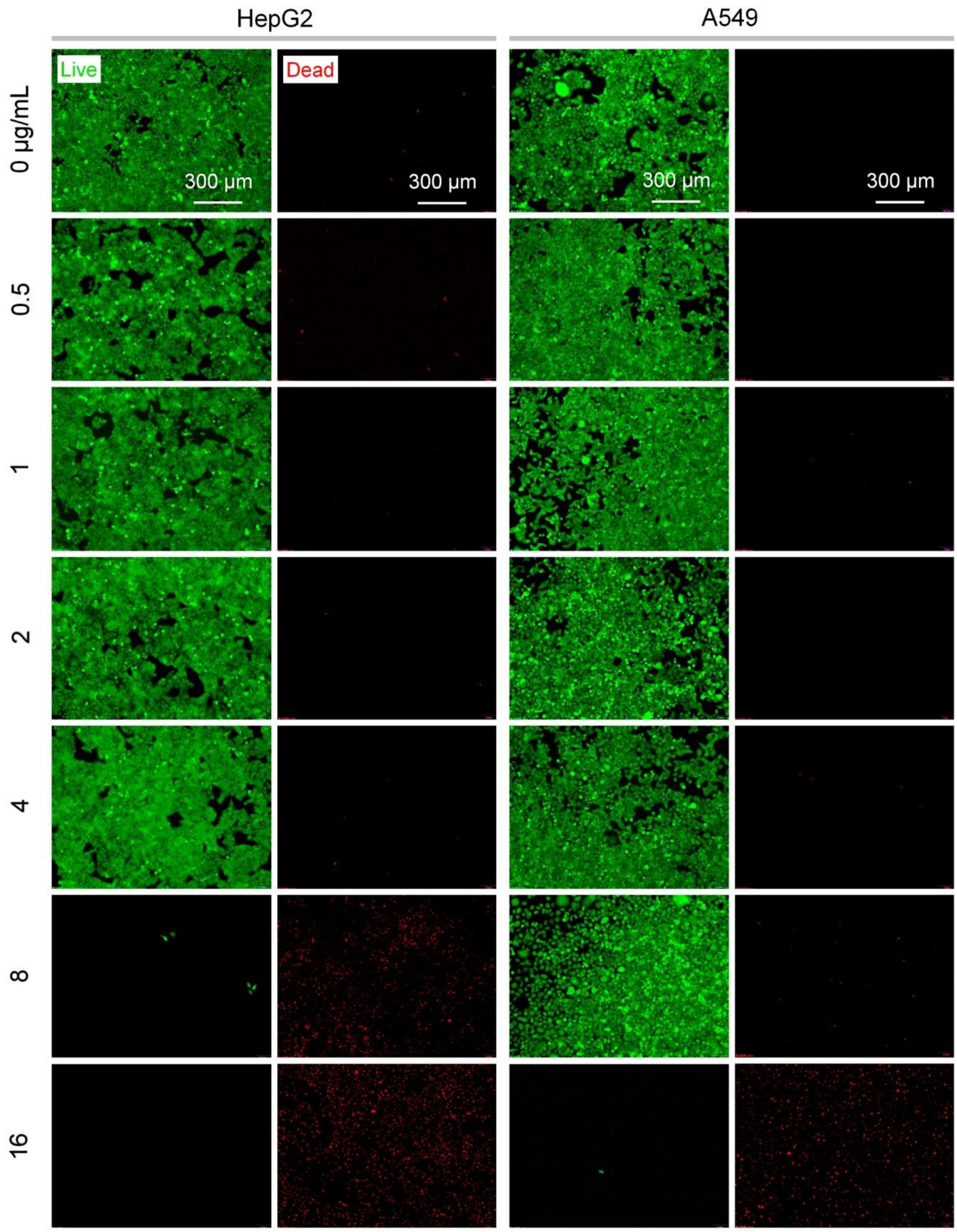

Figure S2. Viability of HepG2 and A549 cells after treatment with different concentrations of Ag-NPs for 24 h. Live/dead (green/red) cells were visualized by FDA/PI staining. Scale bar: 300 μm. The leftmost number was the concentration values of Ag-NPs (μg mL^{-1}).

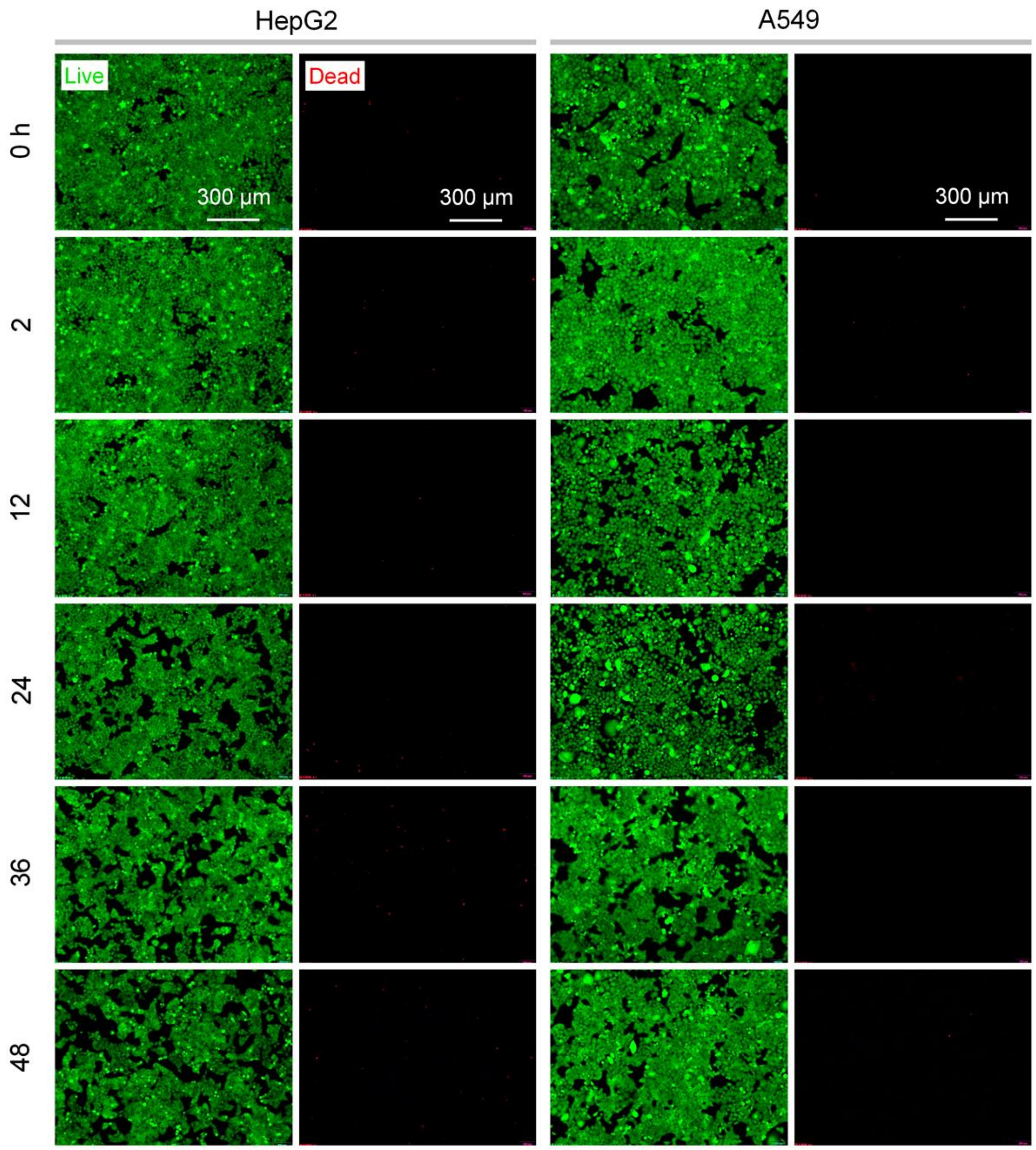

Figure S3. Viability of HepG2 and A549 cells treated with 4 μg mL^{-1} Ag-NPs for various treating times (0, 2, 12, 36, and 48 h). Live/dead (green/red) cells were visualized by FDA/PI staining. Scale bar: 300 μm.

Table S1. Minimum inhibitory concentration (MIC) tests of Ag-NPs against *E. coli, S. aureus and B. subtilis* (n=3)

Ag-NPs concentration (μg mL^{-1})	*E. coli*	*S. aureus*	*B. subtilis*
0 (NC)	−	−	−
0 (PC)	+	+	+
1			+
2	−	+	+
4	−	−	−
8	−	−	−
16	−	−	−
32	−	−	−
64	−	−	−

Note: "−" indicates no bacterial growth, and "+" indicates bacterial growth.

Table S2. Minimum bactericidal concentration (MBC) tests of Ag-NPs against *E. coli, S. aureus and B. subtilis* (n=5)

Ag-NPs concentration (μg mL^{-1})	*E. coli*	*S. aureus*	*B. subtilis*
0	+	+	+
2	+	+	+
4	+	+	+
8	−	−	−

Note: "−" indicates fewer than 5 colonies, and "+" indicates colony counts of ≥5.
